# Classification of drug molecules considering their IC_50 _values using mixed-integer linear programming based hyper-boxes method

**DOI:** 10.1186/1471-2105-9-411

**Published:** 2008-10-03

**Authors:** Pelin Armutlu, Muhittin E Ozdemir, Fadime Uney-Yuksektepe, I Halil Kavakli, Metin Turkay

**Affiliations:** 1 Department of Industrial Engineering, Koç University, Rumelifeneri Yolu, Sariyer, Istanbul 34450, Turkey; 2 Center for Computational Biology and Bioinformatics, Koç University, Rumelifeneri Yolu, Sariyer, Istanbul 34450, Turkey; 3 Department of Chemical and Biological Engineering, Koç University, Rumelifeneri Yolu, Sariyer, Istanbul 34450, Turkey

## Abstract

**Background:**

A priori analysis of the activity of drugs on the target protein by computational approaches can be useful in narrowing down drug candidates for further experimental tests. Currently, there are a large number of computational methods that predict the activity of drugs on proteins. In this study, we approach the activity prediction problem as a classification problem and, we aim to improve the classification accuracy by introducing an algorithm that combines partial least squares regression with mixed-integer programming based hyper-boxes classification method, where drug molecules are classified as low active or high active regarding their binding activity (IC_50 _values) on target proteins. We also aim to determine the most significant molecular descriptors for the drug molecules.

**Results:**

We first apply our approach by analyzing the activities of widely known inhibitor datasets including Acetylcholinesterase (ACHE), Benzodiazepine Receptor (BZR), Dihydrofolate Reductase (DHFR), Cyclooxygenase-2 (COX-2) with known IC_50 _values. The results at this stage proved that our approach consistently gives better classification accuracies compared to 63 other reported classification methods such as SVM, Naïve Bayes, where we were able to predict the experimentally determined IC_50 _values with a worst case accuracy of 96%. To further test applicability of this approach we first created dataset for Cytochrome P450 C17 inhibitors and then predicted their activities with 100% accuracy.

**Conclusion:**

Our results indicate that this approach can be utilized to predict the inhibitory effects of inhibitors based on their molecular descriptors. This approach will not only enhance drug discovery process, but also save time and resources committed.

## Background

At the initial stages of drug discovery and design, there are often millions of candidate drug molecules under consideration. Therefore, the early prediction of activity for drug candidates using computational methods is very important to save time and resources. Due to importance of early prediction of activity of drug candidates on the target protein, a large number of computational methods were developed. QSAR (Quantitative Structure-Activity Relationship) analysis is one of the most widely used methods to relate structure to function. QSAR analysis can be described as the quantitative effort of understanding the correlation between the chemical structure of a molecule and its biological and chemical activities such as biotransformation ability, reaction ability, solubility or target activity[1]. QSAR assumes that structurally similar molecules should have similar activities, which draws attention to the importance of detecting the most significant chemical and structural descriptors of the drug candidates. The drug activity behavior can be predicted using a wide range of descriptors.

Some of the most widely used 3D QSAR methods can be listed as follows: comparative molecular field analysis (CoMFA), comparative molecular similarity indices analysis (CoMSIA), eigenvalue analysis (EVA). In CoMFA, molecular descriptors are calculated and selected by calculating the electrostatic and steric potential energies between a positively charged carbon atom located at each vertex of a rectangular grid and a series of molecules embedded within the grid[2]. The sensitivity to small changes in the alignment of compounds is reduced and hydrogen-bonding and hydrophobic fields are introduced to in CoMSIA[3]. In these methods aligning of the structures is essential, therefore EVA was used due to the fact that methods that are sensitive to 3D structure but do not require superposition were introduced[4]. The generation of descriptors in EVA is based on molecular vibrations, where a normal mode calculation is required to simulate the IR spectrum of a molecule [5].

In this study E-Dragon [6-8], which is a remote version of the DRAGON descriptor calculation program, was used to calculate the molecular descriptors for drugs. It applies the calculation of molecular descriptors developed by Todeschini et. al[9] and provides more than 1,600 molecular descriptors, which are divided into 20 blocks, including atom types, functional group and fragment counts, topological and geometrical descriptors, autocorrelation and information indices, 3D molecular descriptors, molecular properties [6-8]. DRAGON incorporates two steps; the first step eliminates low-variable descriptors, the second step optimizes the descriptor subset using a Q^2^-guided descriptor selection by means of a genetic algorithm using several data analysis methods: Unsupervised Forward Selection (UFS)[10], Associative Neural Network (ASNN)[11,12], Polynomial Neural Network (PNN)[13,14] and Partial Least Squares (PLS) [6-8].

In most studies, Partial Least Squares (PLS)[15] is used to develop QSAR models by reducing the number of attributes in the descriptor set to a small number of attributes correlated with the defined property being modeled.

In our approach, to classify activities of drug compounds, we used the mixed-integer programming (MILP) based hyper-boxes method that takes the molecular descriptors as attributes of the model. The problem of QSAR analysis and the classification of drug candidates are addressed based on their published IC_50 _values by introducing an algorithm that combines PLS regression with mixed-integer linear programming based hyper-boxes classification method. The strength of the algorithm not only comes from combining regression with classification but also the ability to improve the classification accuracies by its iterative approach. The algorithm that links QSAR descriptor model generation with inhibitory activity classification was applied to inhibitors of Acetylcholinesterase (ACHE), Benzodiazepine Receptor (BZR), Dihydrofolate Reductase (DHFR) and Cyclooxygenase-2 (COX-2) and finally for Cytochrome P450 C17 (CYP17).

The comparison of our classification accuracies with the accuracies of the classification methods available in the WEKA data mining package [16] is also made. WEKA contains 63 different classification methods, but here only 16 of those, which had the best classification accuracies for the data sets considered in this paper are discussed. Brief overview of these classifiers is further presented in the Methods section. Our approach outperformed all of the classifiers available in WEKA for each model of the all of the 7 data sets, even reaching 100% accuracy in predicting the activity classification of the inhibitor sets, Ache inhibitors and Cytochrome P450 C17. A total number of 21 QSAR models were built in this study for 7 inhibitor sets, and in 18 of them the accuracy of our methodology exceeded the accuracy of the second best classifier with more than 10%. Through all of the 21 models, the smallest difference in the accuracies is 6.31% and the largest difference is 27.47%.

## Results

To determine the threshold values, which divide the low and high classes, for all datasets the IC_50 _values were statistically analyzed. In this study, we consider 6 datasets, of which IC_50 _values and structures were reported [16-26]. In addition to these datasets we introduced a new dataset for Cytochrome P450 C17 inhibitors that we collected from the literature. Cytochrome P450 C17 is a well-recognized target for prostate cancer treatment, since selective inhibition of the enzyme exerts control over androgen synthesis [27].

After building the descriptor models by e-Dragon [8], three models were constructed during the PLS analysis as: 7, 10 and 15 descriptor models. The reason that we build 3 models with different number of variables is due to the fact that we might come up with insignificant descriptors within one of these models, so that we can replace them by a more significant one from the other models.

The QSAR models with the most significant descriptors, as they were concluded as a result of the initial PLS study for the 7, 10 and 15 attribute models are listed in Table 1 with their R^2 ^values. Table 1 shows the optimal R^2 ^values of our PLS models given by Minitab[28] with predefined number of descriptors from the descriptors calculated by e-Dragon software, and the R^2 ^values of the PLS models calculated by Sutherland et al[26] with the same data sets but different methods and models.

**Table 1:** Comparison of R^2 ^values for PLS models.

Data set	CoMFA*	CoMSIAbasic*	CoMSIAextra*	EVA*	HQSAR*	2D*	2.5D*	e-DragonPLS-7	e-DragonPLS-10	e-DragonPLS-15
AchE	0.88	0.86	0.86	0.96	0.72	0.40	0.38	0.84	0.90	0.95
BZR	0.61	0.62	0.62	0.51	0.64	0.51	0.52	0.51	0.67	0.79
COX-2	0.70	0.69	0.69	0.68	0.70	0.62	0.68	0.53	0.61	0.73
DHFR_RL	0.79	0.76	0.75	0.81	0.81	0.61	0.65	0.42	0.53	0.64
DHFR_PC	N/A	N/A	N/A	N/A	N/A	N/A	N/A	0.44	0.54	0.65
DHFR_TG	N/A	N/A	N/A	N/A	N/A	N/A	N/A	0.40	0.51	0.66
Cytochrome P450 C17	N/A	N/A	N/A	N/A	N/A	N/A	N/A	0.84	0.91	0.95

* PLS results reported by Sutherland et al. [26].

The R^2 ^values shows that, the models we developed with 10 and15 descriptors for Ache BZR and COX-2 are stronger than or at least as strong as the other models reported by Sutherland et al[26] in representing the IC_50 _values in terms of selected descriptors, but our model for DHFR_RL is not as good as the other reported models. High R^2 ^values of Cytochrome P450 C17 models also suggest good prediction of the IC_50 _values and a promising initial model for classification.

It is worth noting that, our study is not simply a regression study, but we develop these regression models in order to use the selected descriptors from this step as attributes for accurate classification.

### Iterations

At the end of the initial runs of the hyper-boxes classification method, classification results are obtained. The next step is the significance analysis and the improvement of the classification accuracies by iterations. The weakest and the strongest descriptors were calculated by significance analysis and, the weakest descriptor in the current model was replaced by the most significant one from other models at each iteration. The classification runs are repeated after each replacement, by MILP based hyper-boxes method. When the classification accuracy is not improved at the end of iteration, the algorithm stops and final results are reported (Table 2).

**Table 2:** Classification Accuracies of each iteration.

		**Iter #0**	**Iter #1**	**Iter #2**	**Iter #3**
**ACHE**	**7 Attributes**	91.89	100.00		
	**10 Attributes**	86.48	89.19	91.89	
	**15 Attributes**	86.05	89.18		
**BZR**	**7 Attributes**	90.90	96.36		
	**10 Attributes**	92.73	94.55		
	**15 Attributes**	90.09	92.73		
**COX-2**	**7 Attributes**	94.39	95.33	97.20	98.13
	**10 Attributes**	91.58	97.20		
	**15 Attributes**	88.78	89.72	90.65	
**DHFR_RL**	**7 Attributes**	94.73	96.99		
	**10 Attributes**	93.98	97.74		
	**15 Attributes**	94.73			
**DHFR_PC**	**7 Attributes**	95.23	96.83	97.62	
	**10 Attributes**	94.44	95.24	98.41	
	**15 Attributes**	92.06	93.65		
**DHFR_TG**	**7 Attributes**	96.24	97.74		
	**10 Attributes**	93.23	93.98	96.24	
	**15 Attributes**	96.24	97.74		
**P450 C17**	**7 Attributes**	86.36	90.00	97.20	100.00
	**10 Attributes**	100.00			
	**15 Attributes**	100.00			

While choosing the weakest descriptor to leave the model, the descriptor with the maximum p-value (failed to reject H_0 _with the greatest error, see methods section for our hypothesis) for one of the high or low classes was selected. The weakest descriptor was replaced by the strongest one. The strongest descriptor defined as the attribute whose maximum p-value for high and low classes is the minimum among the other descriptors.

### Final Classification

As shown in Table 3, we compared the classification accuracies of our model with the results that calculated using all of the classification methods in WEKA. We report only the results of the16 best performing WEKA classifiers. Our method performed better than all of the other classifiers for every model of each dataset. Our integrated approach of regression and classification for Ache and Cytochrome P450 C17 inhibitors datasets displayed an activity prediction accuracy of 100%. The activity of BZR inhibitors was calculated with the accuracy of 96.36%. We were able to predict the activities of COX-2 inhibitors with 98.13% in a 7-descriptor model. In addition, the prediction accuracy of activity of DHFR_RL, DHFR_PC, and DHFR_TG inhibitors were 97.74%, 98.41% and 97.74% respectively. The best performing WEKA classifiers are also highlighted in Table 3.

**Table 3:** Comparison of classification accuracies of best WEKA classifiers with the MILP based hyper-boxes classification.

	**% accuracy**				**% accuracy**		
			
**ACHE**	**7-attribute**	**10-attribute**	**15-attribute**	**BZR**	**7-attribute**	**10-attribute**	**15-attribute**
**MILP based hyper-boxes method**	**100**	**91.89**	**89.19**	**MILP based hyper-boxes method**	**96.36**	**94.55**	**92.73**
Bayes Network	79.28	77.48	78.38	Bayes Network	77.91	77.3	73.62
Naive Bayes	80.18	80.18	81.08	Naive Bayes	80.37	77.91	66.26
Naive Bayes Simple	81.08	80.18	81.98	Naive Bayes Simple	79.14	77.3	68.71
Naive Bayes Updatable	80.18	80.18	81.08	Naive Bayes Updatable	80.37	77.91	66.26
Lojistic	79.28	***84.68***	80.18	Lojistic	***83.44***	80.98	80.98
Multilayer Perceptron	82.88	81.08	81.08	Multilayer Perceptron	79.75	80.98	79.14
SimpleLogistic	***83.78***	82.88	79.28	SimpleLogistic	80.98	***82.82***	79.14
SMO (WEKA SVM)	79.28	80.18	80.18	SMO (WEKA SVM)	79.14	77.91	77.91
IB1	70.27	80.18	77.48	IB1	72.39	74.85	75.46
Ibk	70.27	80.18	77.48	IBk	72.39	74.85	75.46
Logit Boost	82.88	81.08	***82.88***	Logit Boost	78.53	77.3	77.91
Multi Class Classifier	79.28	***84.68***	80.18	Multi Class Classifier	***83.44***	80.98	***80.98***
Threshold Selector	47.75	68.47	60.36	Threshold Selector	78.53	76.69	75.46
LMT	***83.78***	82.88	79.28	LMT	80.98	***82.82***	79.14
RandomForest	80.18	80.18	81.98	RandomForest	77.3	79.75	***80.98***
OneR	81.08	72.97	72.97	OneR	74.85	74.23	79.14

	**% accuracy**				**% accuracy**		
			
**DHFR_TG**	**7-attribute**	**10-attribute**	**15-attribute**	**COX-2**	**7-attribute**	**10-attribute**	**15-attribute**

**MILP based hyper-boxes method**	**97.74**	**96.24**	**97.74**	**MILP based hyper-boxes method**	**98.13**	**97.2**	**90.65**
Bayes Network	77.33	78.09	73.05	Bayes Network	67.2	67.2	66.88
Naive Bayes	76.57	***79.35***	72.54	Naive Bayes	71.66	70.06	64.65
Naive Bayes Simple	75.57	78.84	67	Naive Bayes Simple	72.29	70.06	64.65
Naive Bayes Updatable	76.57	***79.35***	72.54	Naive Bayes Updatable	71.66	70.06	64.65
Lojistic	75.82	78.84	75.57	Lojistic	72.29	70.38	70.06
Multilayer Perceptron	76.32	77.08	75.06	Multilayer Perceptron	***72.61***	72.29	***75.16***
SimpleLogistic	74.56	77.83	75.31	SimpleLogistic	72.29	71.97	68.47
SMO (WEKA SVM)	72.54	79.09	72.54	SMO (WEKA SVM)	71.02	69.43	69.43
IB1	75.31	79.09	75.82	IB1	69.11	71.02	70.06
Ibk	75.31	79.09	75.82	IBk	69.11	71.02	70.06
Logit Boost	77.33	78.34	78.34	Logit Boost	71.66	70.06	70.7
Multi Class Classifier	75.82	78.84	75.57	Multi Class Classifier	72.29	70.38	70.06
Threshold Selector	69.77	74.81	73.55	Threshold Selector	68.47	65.29	64.65
LMT	76.07	76.57	77.83	LMT	71.34	71.02	68.15
RandomForest	***77.58***	79.09	***80.35***	RandomForest	71.97	***74.2***	70.06
OneR	69.77	69.77	70.53	OneR	70.7	70.38	70.06

	**% accuracy**				**% accuracy**		
			
**DHFR_RL**	**7-attribute**	**10-attribute**	**15-attribute**	**DHFR_PC**	**7-attribute**	**10-attribute**	**15-attribute**

**MILP based hyper-boxes method**	**96.99**	**97.74**	**94.73**	**MILP based hyper-boxes method**	**97.62**	**98.41**	**93.65**
Bayes Network	63.72	71.78	70.5	Bayes Network	80.42	80.42	78.04
Naive Bayes	63.97	68.76	71.7	Naive Bayes	82.54	81.48	80.95
Naive Bayes Simple	63.97	67.75	71	Naive Bayes Simple	82.8	79.89	81.22
Naive Bayes Updatable	63.98	68.77	71.78	Naive Bayes Updatable	82.54	81.48	80.95
Lojistic	***69.52***	73.8	78.58	Lojistic	81.75	83.33	81.75
Multilayer Perceptron	62.72	76.57	77.58	Multilayer Perceptron	82.8	82.8	84.13
SimpleLogistic	66.75	73.55	78.33	SimpleLogistic	80.42	***84.13***	81.22
SMO (WEKA SVM)	64.99	73.05	79.59	SMO (WEKA SVM)	82.28	83.33	79.1
IB1	62.97	75.06	81.11	IB1	82.28	80.16	81.75
Ibk	62.97	75.06	***81.11***	IBk	82.28	80.16	81.75
Logit Boost	64.99	75.06	77.33	Logit Boost	83.33	81.48	81.48
Multi Class Classifier	69.52	73.8	78.59	Multi Class Classifier	81.75	83.33	81.75
Threshold Selector	64.99	69.52	78.59	Threshold Selector	83.33	79.1	81.22
LMT	65.24	***77.33***	77.83	LMT	***83.6***	83.07	***85.19***
RandomForest	68.51	77.08	77.83	RandomForest	82.8	80.95	83.07
OneR	61.46	66	62.72	OneR	79.89	79.89	80.16

To verify the reliability of the accuracies given by 10-fold cross validation standard deviations of the classification accuracies were also calculated for each run of MILP based hyper-boxes method. The sensitivity of classification accuracy to the number of descriptors is also examined and the results are reported in Table 4. Small number of descriptors may lead to poor models while a large number of descriptors may lead to inefficient models that incorporate non-informative descriptors for classification. In all of the datasets considered in this paper, this trend is observed from the accuracy values and standard deviation of accuracies for 10-fold cross validation.

**Table 4:** Final average classification accuracies and corresponding standard deviations of classification with 10-fold cross validation with various number of descriptors.

		Average Accuracy	Std. Dev			Average Accuracy	Std. Dev
**ACHE**	4 Attributes	80.83	4.36	**DHFR_RL**	4 Attributes	82.15	2.76
	6 Attributes	83.36	3.67		6 Attributes	91.67	1.86
	7 Attributes	100	0		7 Attributes	96.99	2.14
	8 Attributes	96.36	1.89		8 Attributes	96.64	0.72
	10 Attributes	91.89	2.22		10 Attributes	97.74	0.82
	12 Attributes	86.63	3.28		12 Attributes	97.37	1.33
	15 Attributes	89.18	1.18		15 Attributes	94.73	1.94
	20 Attributes	83.65	3.26		20 Attributes	95.25	3.28
**BZR**	4 Attributes	86.83	1.36	**DHFR_PC**	4 Attributes	81.27	4.72
	6 Attributes	88.36	2.57		6 Attributes	94.48	3.97
	7 Attributes	96.36	2.06		7 Attributes	97.62	2.22
	8 Attributes	93.65	3.83		8 Attributes	96.15	0.82
	10 Attributes	94.55	2.37		10 Attributes	98.41	1.18
	12 Attributes	95.63	1.06		12 Attributes	92.18	2.83
	15 Attributes	92.73	1.46		15 Attributes	93.65	0.98
	20 Attributes	86.25	2.12		20 Attributes	94.25	4.02
**COX-2**	4 Attributes	91.86	3.86	**DHFR_TG**	4 Attributes	84.94	1.47
	6 Attributes	94.36	1.42		6 Attributes	94.03	3.49
	7 Attributes	98.13	1.73		7 Attributes	97.74	1.62
	8 Attributes	97.65	1.23		8 Attributes	96.05	0.72
	10 Attributes	97.2	2.29		10 Attributes	96.24	2.47
	12 Attributes	96.63	2.16		12 Attributes	95.42	1.79
	15 Attributes	90.65	3.06		15 Attributes	97.74	2.78
	20 Attributes	88.06	1.41		20 Attributes	93.5	2.67

### Detailed analysis: Cytochrome P450 C17 inhibitors

We applied our approach to classify activities of drug molecules in a new data set (P450 C17) that is constructed from data in literature [27,29]. The molecular structures and IC50 values for these molecules are given in Additional File 1. This approach may be utilized for the new molecules that inhibit activity of Cytochrome P450 C 17 before channeling them into experiments.

For the 7, 10 and 15 attribute models the selected most significant descriptors as a result of the initial PLS study are listed with R^2 ^values (Table 1) of 0.946, 0.907 and 0.838 respectively.

When the hyper-boxes model was implemented, 10 and the 15-attribute models reached 100% accuracy, by 10-fold cross validation. The 7-attribute model, however, still needed to be improved since the classification results reached an average accuracy of 96.35%. This led us to conclude that there may be some overestimated descriptors actually having low significance in terms of classifying the drug activity. Therefore, significance tests were performed after the preliminary classification runs.

Table 5 shows the *p*-values for the descriptors at each iteration. At iteration 1, C-027 was detected as insignificant since it has the largest *p*-value among the other descriptors. Then from the significance analysis of 10 descriptor model, EEig04x was chosen to replace it, since its maximum *p*-value is the minimum among the other descriptors. After each replacement, the hyper-boxes classification model was built and performed with the new attributes and, average classification accuracy was determined. The runs were stopped after iteration 3 since we reached 100% accuracy. The classification results are reported in Table 6.

**Table 5:** The descriptors leave the 7 descriptor model and the descriptors replacing them.

	**Iteration 1**	**Iteration 2**	**Iteration 3**
Leaving	maxmax1	maxmax2	maxmax3
	*C-027*	*EEig01d*	*Mor22m*
	0.96416	0.9491	0.67855
Entering	minmax1	minmax2	minmax3
	*EEig04x*	*nHAcc*	*Mor14e*
	0.5455	0.5783	0.5946

The results of the final run of hyper-boxes classification for the 7-descriptor model shows that the effect of changing the less significant descriptors with the more significant ones improved the accuracy of the classification from 96.36% to 100%. Since we have reached 100% accuracy in 7-descriptors models, the significant ones may be included in this model among 912-descriptors that are initially calculated by e-DRAGON. Brief explanations of the descriptors can be found in Table 7. [9].

**Table 6:** Comparison of classification accuracies of best WEKA classifiers with MILP based hyper-boxes classification on P450 C17 inhibitors.

	**% accuracy**		
	
**Cytochrome P450 C17**	**7-attribute**	**10-attribute**	**15-attribute**
**MILP based hyper-boxes method**	**100.00**	**100.00**	**100.00**
Bayes Network	***81.25***	***81.25***	***81.25***
Naive Bayes	62.50	71.88	53.13
Naive Bayes Simple	62.50	68.75	50.00
Naive Bayes Updatable	62.50	71.88	53.13
Lojistic	71.88	56.25	62.50
Multilayer Perceptron	62.50	71.88	59.38
SimpleLogistic	75.00	75.00	***81.25***
SMO	***81.25***	***81.25***	***81.25***
IB1	59.38	59.38	***81.25***
IBk	59.38	59.38	62.50
Logit Boost	71.88	62.50	62.50
Multi Class Classifier	71.88	56.25	62.50
Threshold Selector	43.75	40.63	62.50
LMT	75.00	75.00	***81.25***
RandomForest	75.00	68.75	65.63
OneR	75.00	71.88	75.00

**Table 7:** Brief explanation of the most significant descriptors.

**Descriptor**	Brief explanation
**Mor10m**	3D-MoRSE – signal 10/weighted by atomic masses
**DISPp**	d COMMA2 value/weighted by atomic polarizabilities
**Mor14e**	3D-MoRSE – signal 14/weighted by atomic Sanderson electronegativities
**Mor08m**	3D-MoRSE – signal 08/weighted by atomic masses
**nHAcc**	number of acceptor atoms for H-bonds (N. O. F)
**EEig04x**	Eigenvalue 04 from edge adj. matrix weighted by edge degrees
**DISPv**	d COMMA2 value/weighted by atomic van der Waals volumes

## Discussion

Early analysis and estimation of the drug activities by computational methods are widely studied in order to narrow down drug candidates for further experimental tests. The accuracy comparison of our algorithm with other QSAR algorithms suggests that drug activities can be classified with a significantly higher accuracy with the method introduced in this study.

After model building by E-dragon QSAR software, the PLS runs were performed to determine the best model in representing the depended variables (IC_50 _values) in terms of the independent variables (the attributes). The corresponding R^2 ^values were calculated to determine the reliability of the PLS models, where a model with a higher R^2 ^value can be regarded as a more reliable model to proceed to the classification step. The R^2 ^values for the 15, 10 and 7 descriptor models of P450 C17 were obtained by PLS runs and, with a considerable strength in representing the IC_50 _values we accepted the initial models. While the high R^2 ^values of the Ache inhibitor models also were promising on its own to build the classification model, the initial models of BZR and COX-2 inhibitor sets were chosen after the comparison of PLS results with the results reported in the literature as presented in Table 3. For DHFR inhibitors data sets such comparison was not also possible, therefore the models with the best R^2^values in PLS studies were chosen among all other possible models calculated. R^2 ^value directly depends on the values of attributes (the descriptors) and the number of attributes in the corresponding model.

We first applied our iterative algorithm to the large and widely used QSAR data sets in order to validate our methodology. The strength of our algorithm was presented by comparing our classification accuracies with the classification accuracies of 63 WEKA classifiers, on 7 inhibitor sets. The attribute sets prepared as the input for WEKA classifiers were the same as the ones, by which we built the last iteration of our MILP based hyper-boxes classification model. In other words, those were the most significant attributes that we used to develop the final classification models and reached our best accuracies. Our approach outperformed all of the classifiers available in WEKA for each model of the all of the 7 data sets, even reaching 100% accuracy in predicting the activity classification of the inhibitor sets, Ache inhibitors and Cytochrome P450 C17. A total number of 21 QSAR models were built in this study for 7 inhibitor sets, and in 18 of them the accuracy of our methodology exceeded the accuracy of the second best classifier with more than 10%. Through all of the 21 models, the smallest difference in the accuracies is 6.31% and the largest difference is 27.47%.

The higher prediction accuracy of the model not only comes from the choice of initial models by PLS analysis but also the characteristics of MILP based hyper-boxes method. The MILP based hyper-boxes approach allows using more than one hyper-box in order to define a single class [30]. Moreover, this approach deals with problematic and non-problematic instances separately and prevents overlapping of final hyper-boxes [31]. Therefore, these strengths significantly improve the accuracy and efficiency of the MILP based hyper-boxes approach compared to other data classification methods. Data on true positive and false positive rates for accuracy comparison of classifiers for all data sets are given in Additional File 2.

## Conclusion

Drug molecules can be classified as low active or high active based on IC_50 _values. In this study an integrated approach was introduced, which combines the MILP based hyper-boxes method with partial least squares regression to effectively classify the drug candidates. As a result, the most significant molecular descriptors of the drug molecules were also reported. WEKA is used to compare the classification accuracies of the developed model with the classifiers in the WEKA data mining package. The best classification algorithm in WEKA database gave an accuracy of maximum 85% in classifying the activity of drug molecules, through the datasets used in this study.

Our method was applied in order to predict the activities of widely known inhibitor datasets for Acetylcholinesterase (ACHE), Benzodiazepine Receptor (BZR), Dihydrofolate Reductase (DHFR), Cyclooxygenase-2 (COX-2) with known IC50 values. The results suggests that the approach used in this paper results in better classification accuracies compared to other classification methods reported in literature. This approach also applied to Cytochrome P450 C17 inhibitors and their activities were predicted with 100% accuracy.

## Methods

In this paper, we present an integrated approach combining statistical analysis and MILP based hyper-boxes classification method for early prediction of drug behavior targeting Ache, BZR, COX-2, DHFR_TG, DHFR_RL, DHFR_PC, and finally Cytochrome P450 C17.

The approach used in this paper is composed of five main steps. In the first step, molecular structures of the drug candidates is built and optimized the by Marvin Sketch[32]. Then, the molecular descriptors of these drug candidates are obtained using the web server E-Dragon [6-8]. The second step consists of building the regression model using PLS, which will result in selecting the most significant descriptors. Then drug candidates are classified based on the most significant descriptors that are obtained by the previous step, using MILP based hyper-boxes method. This primary classification may result in relatively lower classification accuracy due to the existence of a few insignificant descriptors in the model; therefore, a significance testing analysis is conducted in order to determine the insignificant descriptors that might interfere with our classification accuracy in fourth step. If there are insignificant descriptors in the model we replace the insignificant descriptors with more significant ones; then return to the third step where we classify the drug activities again with the new model that is obtained in step five. After the significance tests if all of the descriptors are significant we build our model with the most significant ones, and report the classification results.

**Figure 1:**
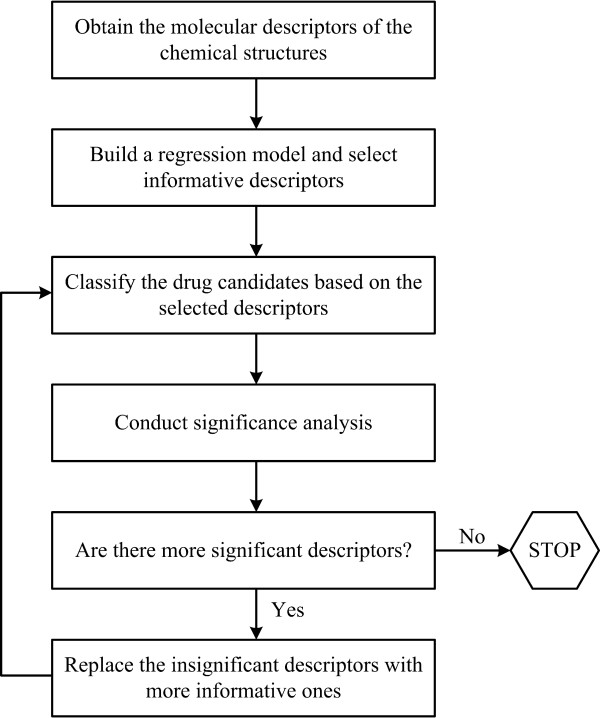
Outline of classification approach.

We use an iterative algorithm such that, some of the steps can be repeated when the significance tests give unsatisfactory results for the selected descriptors of a particular model. Less significant descriptors are replaced with a more significant ones affecting the final classification of the drugs at each iteration, thus improves the success of the study. The outline of our method is given in Figure 1.

### Data sets

We applied our algorithm to widely known QSAR data sets available in literature. Dihydrofolate Reductase (DHFR), Acetylcholinesterase (AchE), Benzodiazepine Receptor (BZR) and Cyclooxygenase-2 (COX-2) inhibitor sets are used for classification. We also introduce a new dataset of Cytochrome P450 C17 inhibitors, which we have derived from the literature and calculated their 3D structures.

**Figure 2:**
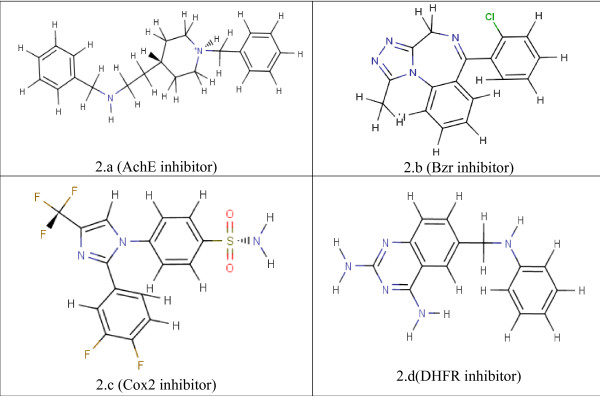
Representative compounds from each QSAR data.

Seven data sets were used for the validation of our methodology by applying the algorithm on these large and known data sets and comparing our classification accuracy on these data sets with the other widely used classifiers available in the WEKA data mining package. Representative compounds from each data set are shown in Figure 2. The experimental IC_50 _values for the dihydrofolate reductase (DHFR) inhibitor set were calculated and reported [16,19,22,26] for the DHFR enzyme from three different species: *P. carinii *(PC), *T. gondii *(TG) and rat liver (RL), where the activity of the DHFR inhibitors to the enzymes from different species differ. Therefore, activities of the inhibitors towards the enzymes from these three species for DHFR inhibitors are studied separately in our study. A set of 397 dihydrofolate reductase inhibitors (DHFR) were used for *P. carinii *DHFR with IC_50 _values from 0.31 nM to 3700 μM, a set of 378 inhibitors were used for *T. gondii *DHFR with values from 0.88 nM to 392 μM and 397 inhibitors were used for rat liver DHFR with values from 0.156 nM to 7470 μM. A set of 111 acetylcholinesterase (AchE) inhibitors were used with experimentally calculated IC_50 _values, reported by within the range of 0.3 nM to 100 μM [23-26]. The data set of the benzodiazepine receptor (BZR) inhibitors consisted of 163 inhibitors, whose IC_50 _values were calculated experimentally from 1.2 nM to 5 μM[20,26]. The 322 molecules of cyclooxygenase-2 (COX2) inhibitor set were derived such that IC_50 _values from 1 nM to 100 μM [17,21,26]. The QSAR sets used in this study were also used in a comparison study of QSAR methods by Sutherland et al[26]. We also compared the R^2 ^values of our 3D descriptor models, which were calculated by the Minitab PLS runs in the first phase of our algorithm, with the reported R^2 ^values by Sutherland et al [27] for several PLS models on the same data sets.

### Structure building and obtaining the descriptor model

As outlined above, in our study the first step is finding molecular descriptors for the drug candidates. Therefore, Marvin Sketch [32] was used to calculate the molecular structures of each drug candidate should be constructed by building their structure and optimize their energy by minimization to determine their confirmation in 3-D space. Next, the optimized 3-D structures are loaded to E-Dragon and molecular descriptors are calculated by using the web server.

E-Dragon suggests many descriptor blocks, each of which contains parameters that describe the characterization of molecules, and the ones that are used in this study can be listed as follows: constitutional descriptors (48), topological descriptors (119), connectivity indices (33), information indices (47), edge adjacency indices (107), topological charge indices (21), geometrical descriptors (74), 3D-MoRSE descriptors (160), functional group counts (154), atom-centered fragments (120), molecular properties (29)[9]. Therefore, the total number of descriptors considered is 912 while building our QSAR descriptor model. PLS[15] is selected for regression analysis because the number of instances is much smaller than the number of attributes (descriptors) by using MINITAB[28]. As we mentioned before, PLS is widely used to develop QSAR models by reducing the number of attributes in the descriptor set to a small number of attributes correlated with the defined property being modeled, which is experimental IC_50 _values in our study.

### Model building with PLS for the selection of the most informative descriptors

The main purpose of the regression analysis is to determine the model that predicts the activity (IC_50_) of the drug candidates in terms of the descriptors. PLS can be referred as an MLR method closely related to principal component regression. Basically, by conducting a PLS study we can predict a set of dependent variables *Y *based on a set of independent variables *X *by MINITAB[28], which gave us the PLS runs automatically based on the upper limit we determined on the number of most significant descriptors. Each PLS run provides a linear model of the dependent variable (IC_50 _values) with respect to the independent variables (most significant descriptors). At this point, the relevant model is built and the most significant descriptors are determined. The next step would be the initial classification of the drugs based on the descriptors. The choice of the significant descriptors by the first PLS runs may not be the most effective ones in classification. Therefore, we perform significance tests on the selected descriptors by the regression analysis to increase the classification accuracies.

### Classification of drug candidates with MILP based hyper-boxes method

The third step is devoted to the classification of drugs; we apply the MILP based hyper-boxes method [30,31] by using the selected descriptors from the previous step.

**Figure 3:**
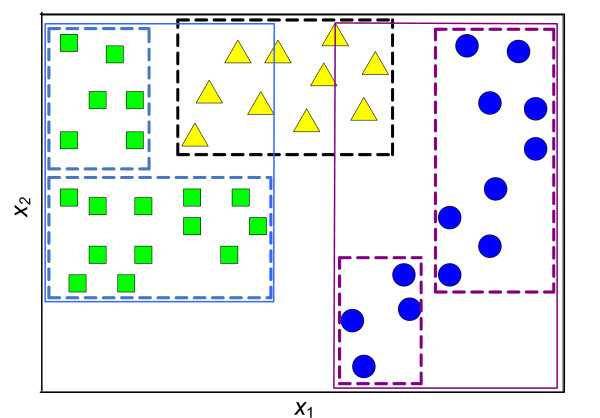
Schematic representation of multi-class data classification using hyper-boxes.

The objective in data classification problems is to assign data points, which are described with certain number of attributes, into predefined classes. The strength of hyper-boxes classification method is from its ability to use more than one hyper-box when defining a class as shown in Figure 3, and this ability prevents overlapping in the classes, which would not be prevented if the classes were defined with a single hyper-box only[30].

The data classification problem is solved in two steps: training step and testing step. In the training step, the boundaries of the classes are formed by the construction of hyper-boxes, where as the effectiveness of the constructed classes are tested in the testing step[30].

The MILP problem for the classification is constructed such that the objective function is the minimization of the misclassifications in the data set with the minimum number of hyper-boxes in the training step. The minimization of the number of hyper-boxes, i.e. the elimination of unnecessary use of hyper-boxes, is enforced by penalizing the existence of a box with a small scalar in the objective function. In the training part the upper and lower bound of each hyper-box also calculated by the data points enclosed in that hyper-box[30].

In the testing step, the data points are assigned to classes by calculating the distance between the data point to the each box, and determining the box that is closest to the data point. Finally, the original and assigned classes of test data points are compared and the effectiveness of the classification is obtained by means of correctly classified instances[30].

Solving the proposed MILP problem to optimality is computationally challenging for large datasets due to the large number of binary variables. Hence, a three-stage decomposition method for obtaining optimal solutions of large data classification problems is developed[31]. Instances that are difficult to classify are identified in the first stage that we refer to as preprocessing. Moreover, seeds are determined for each class to improve the computational efficiency. With greater emphasis given to these observations, a solution to the problem is obtained in the second stage with the modified model. Last, final assignments and intersection eliminations are carried out in the third step[31].

In this paper, we apply this method described above in classifying the activities of drug molecules for the data sets considered. We perform 10-fold cross validation while choosing the training and test sets, where we partition the datasets randomly into 10 subsamples with equal number of members. From these 10 subsamples 9 of them are combined and used as the training set, and the remaining 1 sub sample is used as the test set. Then the classification is performed 10 times with each of the 10 subsamples used exactly once as the test set. Finally, the accuracy of the classification is reported as the average of these 10 classifications.

We classify each of the drug candidates in the test set as having a low or high IC_50 _value. In this iterative study, this classification step is performed several times: first with the initial set of descriptors then using the enhanced set of descriptors derived from significance analysis.

### Significance analysis

In the fourth step, significance tests are performed. After the PLS runs it is possible to conclude a descriptor as significant while it is not in reality and this problem is resolved by conducting significance tests after primary classification. The main idea behind the significance test is as follows: If *Z *is the whole set of drug candidates, assume after the classification it is divided into two classes, A and B. For a successful classification, the variances of descriptor values should be smaller within classes *A *and *B *than it is for the whole population, *Z*.

The equation given below in Eq. 2.1 exhibits the *F *distribution.

(2.1) $$\frac{S_{ij}^{2}/\sigma_{i}^{2}}{S_{k}^{2}/\sigma_{i}^{2}}=S_{ij}^{2}/S_{ik}^{2}=f_{\nu \eta }$$

where, $S_{ij}^{2}$ is the sample variance of values for descriptor *i *for drug set *j*, ν = *n*-1 and η = *m*-1 are degrees of freedom, and *n *is the number of values of descriptor *i *for the drug set *j*, and *m *is the number of values of descriptor *i *for the drug set *k*.

Then the hypothesis testing is performed by the null hypothesis $S_{ij}^{2}=S_{ik}^{2}$, which suggests that the variance of the whole set of drug candidates is equal to the variance of the drugs within the same class. Since the variance of the whole set of drugs should be larger than the variance within the class, we define our alternative hypothesis as: $H_{a}=S_{ij}^{2}≻S_{ik}^{2}$, where j is a member of a whole data set and k is a member of the class. Note that the *p*-value of *f*_vη _in the current should be smaller than the *p*-value of *f*_vη _in the previous model to accept the alternative hypothesis.

### Building the new classification model

This last step is performed when we conclude that there are overestimated descriptors in the model during step four.

Therefore, a total number of 3 models are constructed through regression analysis by selecting 7, 10 and 15 descriptors respectively as representative variables of each model, and the significance analysis is applied to all of the descriptors in these 3 models. If we conclude existence of an insignificant variable in one of these models, we replace them with the ones that are significant in the other models. This adjustment is proved to improve our classification accuracy. When we are replacing the less significant ones, the remaining 880 descriptors that are eliminated during the PLS analysis are ignored, since these 7, 10, and 15 attributes were chosen by the PLS regression analysis and have a proven strength in describing the IC_50 _values. The main purpose of the PLS regression study in fact is eliminating the statistically meaningless features, and provide us with the most meaningful sample space to further work with.

The results obtained by our method are compared with all of the 63 classification methods available in WEKA, and 16 best WEKA classifiers reported with the results obtained by our algorithm in Table 3, with the corresponding classification accuracy. The attributes used in WEKA classifiers are the same descriptors that are found after the significance tests, and 10-fold cross validation was applied to each classifier including our classification method.

WEKA is a powerful data mining tool to use for comparison purposes, since it includes all widely known machine-learning algorithms among its 63 classifiers. The success of these existing machine learning algorithms in binary classification of active and inactive compounds based on their descriptor values were also previously reported[33]. Following is a brief overview of the best performing data classification methods available in WEKA. A Bayesian network[34]*B *= <*N*, *A*, Φ > is a directed acyclic graph <N, A> with a conditional probability distribution attached to each node, collectively represented by Φ. Each node *n *∈ *N *represents a dataset attribute, and each arc *a *∈ *A *between nodes represents a probabilistic dependency. The Naive Bayes classifier assumes that all of the variables are independent of each other, where the classification node is represented as the parent node of all other nodes[35]. Naive Bayes Simple uses the normal distribution for the modelling of the attributes and handle numeric attributes using supervise discretization, where as Naive Bayes Updateable is an incremental version, which processes one instance at a time, and uses a kernel estimator instead of discretization.

The Logistic classifier[35] builds a two-class logistic regression model. It is a statistical regression model, where logistic regression assumes that the log likelihood ratio of class distributions is linear in the observations. The Simple Logistic classifier builds linear logistic regression models based on a single attribute[35]. The model is a generalized model of the ordinary least squares regression model. Multilayer perceptron[35] is a neural network that uses back propagation. The perceptron, which is a processing element, computes a single output, a nonlinear activation function of linear combination of multiple inputs, whose parameters are learned through the training phase. SMO (sequential minimal optimization)[36], also called the WEKA SVM (support vector machine), is a method to train a support vector classifier using polynomial kernels by breaking a large quadratic programming optimization problem into smaller QP optimization problems.

IB1[35] is listed as a lazy classifier, in a sense that it stores the training instances and it does not really do any work until the classification time. IB1 is an instance based learner. It finds the training instance closest in Euclidian distance to the given test instance. IBk is a k-nearest-neighbor classifier that uses the same idea.

Logit Boost[37] uses additive logistic regression. The algorithm can be accelerated by assigning a specific threshold for weights. Multi Class Classifier[38] uses four distinct two-class classification methods for multiclass problems. The Threshold Selector[35], which is a meta learner optimizes the F-measure by selecting a probability threshold on the classifiers output.

Random forest and LMT are decision tree methods. Random Forest generates random trees by collecting ensembles of random trees, where as LMT builds logistic model trees, and uses cross validation to determine the number of iterations while fitting the logistic regression functions at each node. OneR (one rule)[35] builds a one-level decision tree and learns a rule from each attribute and selects the rule having the smallest error rate as the one rule.

## Authors' contributions

PA gathered all data used in this study, made all computational runs, analysis of the results and drafted the paper. MEO performed the annotation of the drug data and helped in the biological analysis of the results. FUY worked on the development of the mixed-integer programming based hyper-boxes method and helped in the use of computer programs for classification. IHK supervised the biological analysis of the input data, interpretation of the results and helped draft the manuscript. MT instigated and guided the research project, supervised the development of the methods used in this project and helped draft the manuscript. All authors read and approved the manuscript.

## Supplementary Material

Additional file 1 Cytochrome P450 C17 inhibitors, their IC50 values and reference IC50 values with ketoconazole. The molecular structures and IC50 values for Cytochrome P450 C17 inhibitors. Click here for file

Additional file 2 True positive and false positive rates for accuracy comparison of classifiers. Data on true positive and false positive rates for accuracy comparison of classifiers for all data sets. Click here for file
