# Supplementary material for: Classification of drug molecules considering their IC50 values using mixed-integer linear programming based hyper-boxes method
Source: BMC Bioinformatics. 2008 Oct 3;9:411. doi: 10.1186/1471-2105-9-411 (PMC2572625; doi:10.1186/1471-2105-9-411)
Supplement: Additional file 1 — Cytochrome P450 C17 inhibitors, their IC50 values and reference IC50 values with ketoconazole. The molecular structures and IC50 values for Cytochrome P450 C17 inhibitors. [file 1471-2105-9-411-S1.doc]

**Additional File 1 - Cytochrome P450 C17 inhibitors, their IC_50_ values and reference IC_50_ values with ketoconazole.**

|  | **Code** | **Structure** | **IC_50_ for 17-20 Lyase** | **Reference** | **Ketoconazole** |
| --- | --- | --- | --- | --- | --- |
| 1 | 10 |  | 5817.4 nM | [29] | 1100 nm |
| 2 | Abiraterone |  | 800 nM | [27] | 1100 nm |
| 3 | BTB13785 |  | 616.5 nM | [27] | 1100 nm |
| 4 | H-1 |  | 450 nM | [27] | 1100 nm |
| 5 | H-2 |  | 320 nM | [27] | 1100 nm |
| 6 | H-3 |  | 1.7 μM | [27] | 1100 nm |
| 7 | H-4 |  | 12.05 μM | [27] | 1100 nm |
| 8 | H-5 |  | 5.9 μM | [27] | 1100 nm |
| 9 | H-6 |  | 628 nM | [27] | 1100 nm |
| 10 | L-5 |  | 225 nM | [27] | 1100 nm |
| 11 | L-39 |  | 1.0 μM | [27] | 1100 nm |
| 12 | NRB03689 |  | 56 nM | [27] | 1100 nm |
| 13 | NRB03731 |  | 178.2 nM | [27] | 1100 nm |
| 14 | NRB03742 |  | 660.25 nM | [27] | 1100 nm |
| 15 | NRB03849 |  | 562 nM | [27] | 1100 nm |
| 16 | VN/63-1 |  | 800 nM | [27] | 1100 nm |
| 17 | VN/85-1 |  | 50 nM | [27] | 1100 nm |
| 18 | VN/87-1 |  | 377.3 nM | [27] | 1100 nm |
| 19 | VN/90-1 |  | 3.7 μM | [27] | 1100 nm |
| 20 | VN/95-1 |  | 2.6 μM | [27] | 1100 nm |
| 21 | VN/96-1 |  | 1.4 μM | [27] | 1100 nm |
| 22 | VN/107-1 |  | 1.2 μM | [27] | 1100 nm |
| 23 | VN/108-1 |  | 150 nM | [27] | 1100 nm |
| 24 | VN/109-1 |  | 386.9 nM | [27] | 1100 nm |
| 25 | VN/124-1 |  | 300 nM | [27] | 1100 nm |
| 26 | VN/125-1 |  | 915 nM | [27] | 1100 nm |
| 27 | VN/128-1 |  | 1.25 nM | [27] | 1100 nm |
| 28 | VN/DN/4-1 |  | 3.81 μM | [27] | 1100 nm |
| 29 | VN/DN/5-1 |  | 500 nM | [27] | 1100 nm |
| 30 | Xanthene-based 1 |  | 42 nM | [27] | 1100 nm |
| 31 | Xanthene-based 2 |  | 220 nM | [27] | 1100 nm |
| 32 | Xanthene-based 3 |  | 130 nM | [27] | 1100 nm |
