# Supplementary material for: Classification of drug molecules considering their IC50 values using mixed-integer linear programming based hyper-boxes method
Source: BMC Bioinformatics. 2008 Oct 3;9:411. doi: 10.1186/1471-2105-9-411 (PMC2572625; doi:10.1186/1471-2105-9-411)
Supplement: Additional file 2 — True positive and false positive rates for accuracy comparison of classifiers. Data on true positive and false positive rates for accuracy comparison of classifiers for all data sets. [file 1471-2105-9-411-S2.doc]

**Additional File 2 – True positive and false positive rates for accuracy comparison of classifiers**

|  |  | **7-attribute** | | **10-attribute** | | **15-attribute** | |
| --- | --- | --- | --- | --- | --- | --- | --- |
| **ACHE** | **Class** | **TP Rate** | **FP Rate** | **TP Rate** | **FP Rate** | **TP Rate** | **FP Rate** |
| **MILP based** | **LowIC50** | **1.000** | **0.000** | **0.966** | **0.261** | **0.943** | **0.304** |
| **hyper-boxes** | **HighIC50** | **1.000** | **0.000** | **0.739** | **0.034** | **0.696** | **0.057** |
| Bayes Network | LowIC50 | 0.989 | 0.957 | 0.977 | 1.000 | 0.977 | 0.957 |
|  | HighIC50 | 0.043 | 0.011 | 0.000 | 0.023 | 0.043 | 0.023 |
| Naive Bayes | LowIC50 | 0.932 | 0.696 | 0.932 | 0.696 | 0.920 | 0.609 |
|  | HighIC50 | 0.304 | 0.068 | 0.304 | 0.068 | 0.391 | 0.080 |
| Naive Bayes | LowIC50 | 0.943 | 0.696 | 0.932 | 0.696 | 0.932 | 0.609 |
| Simple | HighIC50 | 0.304 | 0.057 | 0.304 | 0.068 | 0.391 | 0.068 |
| Naive Bayes | LowIC50 | 0.932 | 0.696 | 0.932 | 0.696 | 0.920 | 0.609 |
| Updatable | HighIC50 | 0.304 | 0.068 | 0.304 | 0.068 | 0.391 | 0.080 |
| Lojistic | LowIC50 | 0.932 | 0.739 | 0.943 | 0.522 | 0.886 | 0.522 |
|  | HighIC50 | 0.261 | 0.068 | 0.478 | 0.057 | 0.478 | 0.114 |
| Multilayer | LowIC50 | 0.977 | 0.739 | 0.898 | 0.522 | 0.909 | 0.565 |
| Perceptron | HighIC50 | 0.261 | 0.023 | 0.478 | 0.102 | 0.435 | 0.091 |
| SimpleLogistic | LowIC50 | 0.989 | 0.739 | 0.966 | 0.696 | 0.955 | 0.826 |
|  | HighIC50 | 0.261 | 0.011 | 0.304 | 0.034 | 0.174 | 0.045 |
| SMO | LowIC50 | 1.000 | 1.000 | 1.000 | 0.957 | 0.989 | 0.913 |
| (WEKA SVM) | HighIC50 | 0.000 | 0.000 | 0.043 | 0.000 | 0.087 | 0.011 |
| IB1 | LowIC50 | 0.807 | 0.696 | 0.875 | 0.478 | 0.875 | 0.609 |
|  | HighIC50 | 0.304 | 0.193 | 0.522 | 0.125 | 0.391 | 0.125 |
| IBk | LowIC50 | 0.807 | 0.696 | 0.875 | 0.478 | 0.875 | 0.609 |
|  | HighIC50 | 0.304 | 0.193 | 0.522 | 0.125 | 0.391 | 0.125 |
| Logit Boost | LowIC50 | 0.955 | 0.652 | 0.943 | 0.696 | 0.932 | 0.565 |
|  | HighIC50 | 0.348 | 0.045 | 0.304 | 0.057 | 0.435 | 0.068 |
| Multi Class | LowIC50 | 0.932 | 0.739 | 0.943 | 0.522 | 0.886 | 0.522 |
| Classifier | HighIC50 | 0.261 | 0.068 | 0.478 | 0.057 | 0.478 | 0.114 |
| Threshold | LowIC50 | 0.409 | 0.261 | 0.670 | 0.261 | 0.580 | 0.304 |
| Selector | HighIC50 | 0.739 | 0.591 | 0.739 | 0.330 | 0.696 | 0.420 |
| LMT | LowIC50 | 0.989 | 0.739 | 0.966 | 0.696 | 0.955 | 0.826 |
|  | HighIC50 | 0.261 | 0.011 | 0.304 | 0.034 | 0.174 | 0.045 |
| RandomForest | LowIC50 | 0.943 | 0.739 | 0.977 | 0.870 | 0.977 | 0.783 |
|  | HighIC50 | 0.261 | 0.057 | 0.130 | 0.023 | 0.217 | 0.023 |
| OneR | LowIC50 | 0.943 | 0.739 | 0.909 | 0.957 | 0.875 | 0.826 |
|  | HighIC50 | 0.261 | 0.057 | 0.043 | 0.091 | 0.174 | 0.125 |

|  |  | **7-attribute** | | **10-attribute** | | **15-attribute** | |
| --- | --- | --- | --- | --- | --- | --- | --- |
| **BZR** | **Class** | **TP Rate** | **FP Rate** | **TP Rate** | **FP Rate** | **TP Rate** | **FP Rate** |
| **MILP based** | **LowIC50** | **1.000** | **0.167** | **0.992** | **0.222** | **0.984** | **0.278** |
| **hyper-boxes** | **HighIC50** | **0.833** | **0.000** | **0.778** | **0.008** | **0.722** | **0.016** |
| Bayes Network | LowIC50 | 1.000 | 1.000 | 0.976 | 0.944 | 0.850 | 0.667 |
|  | HighIC50 | 0.000 | 0.000 | 0.056 | 0.024 | 0.333 | 0.150 |
| Naive Bayes | LowIC50 | 0.929 | 0.639 | 0.929 | 0.750 | 0.685 | 0.417 |
|  | HighIC50 | 0.361 | 0.071 | 0.250 | 0.071 | 0.583 | 0.315 |
| Naive Bayes | LowIC50 | 0.929 | 0.694 | 0.929 | 0.778 | 0.709 | 0.389 |
| Simple | HighIC50 | 0.306 | 0.071 | 0.222 | 0.071 | 0.611 | 0.291 |
| Naive Bayes | LowIC50 | 0.929 | 0.639 | 0.929 | 0.750 | 0.685 | 0.417 |
| Updatable | HighIC50 | 0.361 | 0.071 | 0.250 | 0.071 | 0.583 | 0.315 |
| Lojistic | LowIC50 | 0.953 | 0.583 | 0.937 | 0.639 | 0.906 | 0.528 |
|  | HighIC50 | 0.417 | 0.047 | 0.361 | 0.063 | 0.472 | 0.094 |
| Multilayer | LowIC50 | 0.913 | 0.611 | 0.882 | 0.444 | 0.913 | 0.639 |
| Perceptron | HighIC50 | 0.389 | 0.087 | 0.556 | 0.118 | 0.361 | 0.087 |
| SimpleLogistic | LowIC50 | 0.969 | 0.750 | 0.969 | 0.667 | 0.913 | 0.639 |
|  | HighIC50 | 0.250 | 0.031 | 0.333 | 0.031 | 0.361 | 0.087 |
| SMO | LowIC50 | 1.000 | 0.944 | 1.000 | 1.000 | 1.000 | 1.000 |
| (WEKA SVM) | HighIC50 | 0.056 | 0.000 | 0.000 | 0.000 | 0.000 | 0.000 |
| IB1 | LowIC50 | 0.827 | 0.639 | 0.866 | 0.667 | 0.874 | 0.667 |
|  | HighIC50 | 0.361 | 0.173 | 0.333 | 0.134 | 0.333 | 0.126 |
| IBk | LowIC50 | 0.827 | 0.639 | 0.866 | 0.667 | 0.874 | 0.667 |
|  | HighIC50 | 0.361 | 0.173 | 0.333 | 0.134 | 0.333 | 0.126 |
| Logit Boost | LowIC50 | 0.921 | 0.694 | 0.921 | 0.750 | 0.898 | 0.639 |
|  | HighIC50 | 0.306 | 0.079 | 0.250 | 0.079 | 0.361 | 0.102 |
| Multi Class | LowIC50 | 0.953 | 0.583 | 0.937 | 0.639 | 0.906 | 0.528 |
| Classifier | HighIC50 | 0.417 | 0.047 | 0.361 | 0.063 | 0.472 | 0.094 |
| Threshold | LowIC50 | 0.882 | 0.556 | 0.858 | 0.556 | 0.819 | 0.472 |
| Selector | HighIC50 | 0.444 | 0.118 | 0.444 | 0.142 | 0.528 | 0.181 |
| LMT | LowIC50 | 0.969 | 0.750 | 0.961 | 0.639 | 0.906 | 0.611 |
|  | HighIC50 | 0.250 | 0.031 | 0.361 | 0.039 | 0.389 | 0.094 |
| RandomForest | LowIC50 | 0.929 | 0.778 | 0.953 | 0.750 | 0.953 | 0.694 |
|  | HighIC50 | 0.222 | 0.071 | 0.250 | 0.047 | 0.306 | 0.047 |
| OneR | LowIC50 | 0.945 | 0.944 | 0.945 | 0.972 | 0.906 | 0.611 |
|  | HighIC50 | 0.056 | 0.055 | 0.028 | 0.055 | 0.389 | 0.094 |

|  |  | **7-attribute** | | **10-attribute** | | **15-attribute** | |
| --- | --- | --- | --- | --- | --- | --- | --- |
| **COX-2** | **Class** | **TP Rate** | **FP Rate** | **TP Rate** | **FP Rate** | **TP Rate** | **FP Rate** |
| **MILP based** | **LowIC50** | **0.978** | **0.015** | **0.961** | **0.015** | **0.860** | **0.037** |
| **hyper-boxes** | **HighIC50** | **0.985** | **0.022** | **0.985** | **0.039** | **0.963** | **0.140** |
| Bayes Network | LowIC50 | 0.882 | 0.603 | 0.888 | 0.610 | 0.826 | 0.537 |
|  | HighIC50 | 0.397 | 0.118 | 0.390 | 0.112 | 0.463 | 0.174 |
| Naive Bayes | LowIC50 | 0.871 | 0.485 | 0.860 | 0.507 | 0.618 | 0.316 |
|  | HighIC50 | 0.515 | 0.129 | 0.493 | 0.140 | 0.684 | 0.382 |
| Naive Bayes | LowIC50 | 0.882 | 0.485 | 0.860 | 0.507 | 0.618 | 0.316 |
| Simple | HighIC50 | 0.515 | 0.118 | 0.493 | 0.140 | 0.684 | 0.382 |
| Naive Bayes | LowIC50 | 0.871 | 0.485 | 0.860 | 0.507 | 0.618 | 0.316 |
| Updatable | HighIC50 | 0.515 | 0.129 | 0.493 | 0.140 | 0.684 | 0.382 |
| Lojistic | LowIC50 | 0.809 | 0.390 | 0.781 | 0.397 | 0.764 | 0.382 |
|  | HighIC50 | 0.610 | 0.191 | 0.603 | 0.219 | 0.618 | 0.236 |
| Multilayer | LowIC50 | 0.820 | 0.397 | 0.798 | 0.375 | 0.837 | 0.360 |
| Perceptron | HighIC50 | 0.603 | 0.180 | 0.625 | 0.202 | 0.640 | 0.163 |
| SimpleLogistic | LowIC50 | 0.826 | 0.412 | 0.820 | 0.412 | 0.809 | 0.478 |
|  | HighIC50 | 0.588 | 0.174 | 0.588 | 0.180 | 0.522 | 0.191 |
| SMO | LowIC50 | 0.820 | 0.434 | 0.837 | 0.493 | 0.798 | 0.441 |
| (WEKA SVM) | HighIC50 | 0.566 | 0.180 | 0.507 | 0.163 | 0.559 | 0.202 |
| IB1 | LowIC50 | 0.747 | 0.382 | 0.775 | 0.375 | 0.758 | 0.375 |
|  | HighIC50 | 0.618 | 0.253 | 0.625 | 0.225 | 0.625 | 0.242 |
| IBk | LowIC50 | 0.747 | 0.382 | 0.775 | 0.375 | 0.758 | 0.375 |
|  | HighIC50 | 0.618 | 0.253 | 0.625 | 0.225 | 0.625 | 0.242 |
| Logit Boost | LowIC50 | 0.798 | 0.390 | 0.803 | 0.434 | 0.809 | 0.426 |
|  | HighIC50 | 0.610 | 0.202 | 0.566 | 0.197 | 0.574 | 0.191 |
| Multi Class | LowIC50 | 0.809 | 0.390 | 0.781 | 0.397 | 0.764 | 0.382 |
| Classifier | HighIC50 | 0.610 | 0.191 | 0.603 | 0.219 | 0.618 | 0.236 |
| Threshold | LowIC50 | 0.635 | 0.250 | 0.596 | 0.272 | 0.573 | 0.257 |
| Selector | HighIC50 | 0.750 | 0.365 | 0.728 | 0.404 | 0.743 | 0.427 |
| LMT | LowIC50 | 0.837 | 0.449 | 0.848 | 0.471 | 0.787 | 0.456 |
|  | HighIC50 | 0.551 | 0.163 | 0.529 | 0.152 | 0.544 | 0.213 |
| RandomForest | LowIC50 | 0.826 | 0.419 | 0.854 | 0.404 | 0.820 | 0.456 |
|  | HighIC50 | 0.581 | 0.174 | 0.596 | 0.146 | 0.544 | 0.180 |
| OneR | LowIC50 | 0.831 | 0.456 | 0.848 | 0.485 | 0.837 | 0.478 |
|  | HighIC50 | 0.544 | 0.169 | 0.515 | 0.152 | 0.522 | 0.163 |

|  |  | **7-attribute** | | **10-attribute** | | **15-attribute** | |
| --- | --- | --- | --- | --- | --- | --- | --- |
| **DHFR_RL** | **Class** | **TP Rate** | **FP Rate** | **TP Rate** | **FP Rate** | **TP Rate** | **FP Rate** |
| **MILP based** | **LowIC50** | **0.985** | **0.046** | **0.985** | **0.031** | **0.960** | **0.066** |
| **hyper-boxes** | **HighIC50** | **0.954** | **0.015** | **0.969** | **0.015** | **0.934** | **0.040** |
| Bayes Network | LowIC50 | 0.736 | 0.464 | 0.781 | 0.347 | 0.741 | 0.332 |
|  | HighIC50 | 0.536 | 0.264 | 0.653 | 0.219 | 0.668 | 0.259 |
| Naive Bayes | LowIC50 | 0.716 | 0.439 | 0.711 | 0.337 | 0.706 | 0.270 |
|  | HighIC50 | 0.561 | 0.284 | 0.663 | 0.289 | 0.730 | 0.294 |
| Naive Bayes | LowIC50 | 0.726 | 0.449 | 0.706 | 0.352 | 0.706 | 0.286 |
| Simple | HighIC50 | 0.551 | 0.274 | 0.648 | 0.294 | 0.714 | 0.294 |
| Naive Bayes | LowIC50 | 0.716 | 0.439 | 0.711 | 0.337 | 0.706 | 0.270 |
| Updatable | HighIC50 | 0.561 | 0.284 | 0.663 | 0.289 | 0.730 | 0.294 |
| Lojistic | LowIC50 | 0.711 | 0.321 | 0.741 | 0.265 | 0.801 | 0.230 |
|  | HighIC50 | 0.679 | 0.289 | 0.735 | 0.259 | 0.770 | 0.199 |
| Multilayer | LowIC50 | 0.716 | 0.464 | 0.801 | 0.270 | 0.756 | 0.204 |
| Perceptron | HighIC50 | 0.536 | 0.284 | 0.730 | 0.199 | 0.796 | 0.244 |
| SimpleLogistic | LowIC50 | 0.672 | 0.337 | 0.741 | 0.270 | 0.796 | 0.230 |
|  | HighIC50 | 0.663 | 0.328 | 0.730 | 0.259 | 0.770 | 0.204 |
| SMO | LowIC50 | 0.682 | 0.383 | 0.721 | 0.260 | 0.841 | 0.250 |
| (WEKA SVM) | HighIC50 | 0.617 | 0.318 | 0.740 | 0.279 | 0.750 | 0.159 |
| IB1 | LowIC50 | 0.647 | 0.388 | 0.771 | 0.270 | 0.831 | 0.209 |
|  | HighIC50 | 0.612 | 0.353 | 0.730 | 0.229 | 0.791 | 0.169 |
| IBk | LowIC50 | 0.647 | 0.388 | 0.771 | 0.270 | 0.831 | 0.209 |
|  | HighIC50 | 0.612 | 0.353 | 0.730 | 0.229 | 0.791 | 0.169 |
| Logit Boost | LowIC50 | 0.741 | 0.444 | 0.741 | 0.240 | 0.771 | 0.224 |
|  | HighIC50 | 0.556 | 0.259 | 0.760 | 0.259 | 0.776 | 0.229 |
| Multi Class | LowIC50 | 0.711 | 0.321 | 0.741 | 0.265 | 0.801 | 0.230 |
| Classifier | HighIC50 | 0.679 | 0.289 | 0.735 | 0.259 | 0.770 | 0.199 |
| Threshold | LowIC50 | 0.493 | 0.189 | 0.557 | 0.163 | 0.761 | 0.189 |
| Selector | HighIC50 | 0.811 | 0.507 | 0.837 | 0.443 | 0.811 | 0.239 |
| LMT | LowIC50 | 0.662 | 0.357 | 0.776 | 0.230 | 0.796 | 0.240 |
|  | HighIC50 | 0.643 | 0.338 | 0.770 | 0.224 | 0.760 | 0.204 |
| RandomForest | LowIC50 | 0.672 | 0.444 | 0.831 | 0.291 | 0.826 | 0.270 |
|  | HighIC50 | 0.556 | 0.328 | 0.709 | 0.169 | 0.730 | 0.174 |
| OneR | LowIC50 | 0.672 | 0.444 | 0.667 | 0.347 | 0.652 | 0.398 |
|  | HighIC50 | 0.556 | 0.328 | 0.653 | 0.333 | 0.602 | 0.348 |

|  |  | **7-attribute** | | **10-attribute** | | **15-attribute** | |
| --- | --- | --- | --- | --- | --- | --- | --- |
| **DHFR_PC** | **Class** | **TP Rate** | **FP Rate** | **TP Rate** | **FP Rate** | **TP Rate** | **FP Rate** |
| **MILP based** | **LowIC50** | **0.975** | **0.020** | **0.993** | **0.041** | **0.979** | **0.184** |
| **hyper-boxes** | **HighIC50** | **0.980** | **0.025** | **0.959** | **0.007** | **0.816** | **0.021** |
| Bayes Network | LowIC50 | 0.882 | 0.418 | 0.882 | 0.418 | 0.850 | 0.418 |
|  | HighIC50 | 0.582 | 0.118 | 0.582 | 0.118 | 0.582 | 0.150 |
| Naive Bayes | LowIC50 | 0.868 | 0.296 | 0.857 | 0.306 | 0.861 | 0.337 |
|  | HighIC50 | 0.704 | 0.132 | 0.694 | 0.143 | 0.663 | 0.139 |
| Naive Bayes | LowIC50 | 0.868 | 0.286 | 0.857 | 0.367 | 0.861 | 0.327 |
| Simple | HighIC50 | 0.714 | 0.132 | 0.633 | 0.143 | 0.673 | 0.139 |
| Naive Bayes | LowIC50 | 0.868 | 0.296 | 0.857 | 0.306 | 0.861 | 0.337 |
| Updatable | HighIC50 | 0.704 | 0.132 | 0.694 | 0.143 | 0.663 | 0.139 |
| Lojistic | LowIC50 | 0.889 | 0.388 | 0.893 | 0.337 | 0.904 | 0.429 |
|  | HighIC50 | 0.612 | 0.111 | 0.663 | 0.107 | 0.571 | 0.096 |
| Multilayer | LowIC50 | 0.875 | 0.306 | 0.871 | 0.296 | 0.886 | 0.286 |
| Perceptron | HighIC50 | 0.694 | 0.125 | 0.704 | 0.129 | 0.714 | 0.114 |
| SimpleLogistic | LowIC50 | 0.882 | 0.418 | 0.900 | 0.327 | 0.904 | 0.449 |
|  | HighIC50 | 0.582 | 0.118 | 0.673 | 0.100 | 0.551 | 0.096 |
| SMO | LowIC50 | 0.864 | 0.296 | 0.857 | 0.235 | 0.875 | 0.449 |
| (WEKA SVM) | HighIC50 | 0.704 | 0.136 | 0.765 | 0.143 | 0.551 | 0.125 |
| IB1 | LowIC50 | 0.879 | 0.337 | 0.871 | 0.398 | 0.893 | 0.398 |
|  | HighIC50 | 0.663 | 0.121 | 0.602 | 0.129 | 0.602 | 0.107 |
| IBk | LowIC50 | 0.879 | 0.337 | 0.871 | 0.398 | 0.893 | 0.398 |
|  | HighIC50 | 0.663 | 0.121 | 0.602 | 0.129 | 0.602 | 0.107 |
| Logit Boost | LowIC50 | 0.900 | 0.357 | 0.904 | 0.439 | 0.889 | 0.398 |
|  | HighIC50 | 0.643 | 0.100 | 0.561 | 0.096 | 0.602 | 0.111 |
| Multi Class | LowIC50 | 0.889 | 0.388 | 0.893 | 0.337 | 0.904 | 0.429 |
| Classifier | HighIC50 | 0.612 | 0.111 | 0.663 | 0.107 | 0.571 | 0.096 |
| Threshold | LowIC50 | 0.850 | 0.214 | 0.800 | 0.235 | 0.829 | 0.235 |
| Selector | HighIC50 | 0.786 | 0.150 | 0.765 | 0.200 | 0.765 | 0.171 |
| LMT | LowIC50 | 0.900 | 0.347 | 0.882 | 0.316 | 0.896 | 0.276 |
|  | HighIC50 | 0.653 | 0.100 | 0.684 | 0.118 | 0.724 | 0.104 |
| RandomForest | LowIC50 | 0.900 | 0.378 | 0.900 | 0.449 | 0.921 | 0.429 |
|  | HighIC50 | 0.622 | 0.100 | 0.551 | 0.100 | 0.571 | 0.079 |
| OneR | LowIC50 | 0.893 | 0.469 | 0.893 | 0.469 | 0.893 | 0.459 |
|  | HighIC50 | 0.531 | 0.107 | 0.531 | 0.107 | 0.541 | 0.107 |

|  |  | **7-attribute** | | **10-attribute** | | **15-attribute** | |
| --- | --- | --- | --- | --- | --- | --- | --- |
| **DHFR_TG** | **Class** | **TP Rate** | **FP Rate** | **TP Rate** | **FP Rate** | **TP Rate** | **FP Rate** |
| **MILP based** | **LowIC50** | **0.977** | **0.023** | **0.985** | **0.083** | **0.977** | **0.023** |
| **hyper-boxes** | **HighIC50** | **0.977** | **0.023** | **0.917** | **0.015** | **0.977** | **0.023** |
| Bayes Network | LowIC50 | 0.906 | 0.492 | 0.860 | 0.379 | 0.891 | 0.591 |
|  | HighIC50 | 0.508 | 0.094 | 0.621 | 0.140 | 0.409 | 0.109 |
| Naive Bayes | LowIC50 | 0.875 | 0.455 | 0.891 | 0.402 | 0.883 | 0.591 |
|  | HighIC50 | 0.545 | 0.125 | 0.598 | 0.109 | 0.409 | 0.117 |
| Naive Bayes | LowIC50 | 0.857 | 0.447 | 0.879 | 0.394 | 0.709 | 0.409 |
| Simple | HighIC50 | 0.553 | 0.143 | 0.606 | 0.121 | 0.591 | 0.291 |
| Naive Bayes | LowIC50 | 0.875 | 0.455 | 0.891 | 0.402 | 0.883 | 0.591 |
| Updatable | HighIC50 | 0.545 | 0.125 | 0.598 | 0.109 | 0.409 | 0.117 |
| Lojistic | LowIC50 | 0.898 | 0.523 | 0.887 | 0.409 | 0.913 | 0.561 |
|  | HighIC50 | 0.477 | 0.102 | 0.591 | 0.113 | 0.439 | 0.087 |
| Multilayer | LowIC50 | 0.860 | 0.432 | 0.838 | 0.364 | 0.811 | 0.371 |
| Perceptron | HighIC50 | 0.568 | 0.140 | 0.636 | 0.162 | 0.629 | 0.189 |
| SimpleLogistic | LowIC50 | 0.891 | 0.545 | 0.906 | 0.477 | 0.932 | 0.606 |
|  | HighIC50 | 0.455 | 0.109 | 0.523 | 0.094 | 0.394 | 0.068 |
| SMO | LowIC50 | 0.921 | 0.667 | 0.925 | 0.477 | 0.947 | 0.720 |
| (WEKA SVM) | HighIC50 | 0.333 | 0.079 | 0.523 | 0.075 | 0.280 | 0.053 |
| IB1 | LowIC50 | 0.845 | 0.432 | 0.864 | 0.356 | 0.838 | 0.402 |
|  | HighIC50 | 0.568 | 0.155 | 0.644 | 0.136 | 0.598 | 0.162 |
| IBk | LowIC50 | 0.845 | 0.432 | 0.864 | 0.356 | 0.838 | 0.402 |
|  | HighIC50 | 0.568 | 0.155 | 0.644 | 0.136 | 0.598 | 0.162 |
| Logit Boost | LowIC50 | 0.872 | 0.424 | 0.894 | 0.439 | 0.913 | 0.477 |
|  | HighIC50 | 0.576 | 0.128 | 0.561 | 0.106 | 0.523 | 0.087 |
| Multi Class | LowIC50 | 0.898 | 0.523 | 0.887 | 0.409 | 0.913 | 0.561 |
| Classifier | HighIC50 | 0.477 | 0.102 | 0.591 | 0.113 | 0.439 | 0.087 |
| Threshold | LowIC50 | 0.702 | 0.311 | 0.774 | 0.303 | 0.796 | 0.386 |
| Selector | HighIC50 | 0.689 | 0.298 | 0.697 | 0.226 | 0.614 | 0.204 |
| LMT | LowIC50 | 0.883 | 0.485 | 0.868 | 0.439 | 0.898 | 0.462 |
|  | HighIC50 | 0.515 | 0.117 | 0.561 | 0.132 | 0.538 | 0.102 |
| RandomForest | LowIC50 | 0.879 | 0.432 | 0.925 | 0.477 | 0.917 | 0.424 |
|  | HighIC50 | 0.568 | 0.121 | 0.523 | 0.075 | 0.576 | 0.083 |
| OneR | LowIC50 | 0.838 | 0.583 | 0.838 | 0.583 | 0.872 | 0.629 |
|  | HighIC50 | 0.417 | 0.162 | 0.417 | 0.162 | 0.371 | 0.128 |

| **Cytochrome** |  | **7-attribute** | | **10-attribute** | | **15-attribute** | |
| --- | --- | --- | --- | --- | --- | --- | --- |
| **P450 C17** | **Class** | **TP Rate** | **FP Rate** | **TP Rate** | **FP Rate** | **TP Rate** | **FP Rate** |
| **MILP based** | **LowIC50** | **1.000** | **0.000** | **1.000** | **0.000** | **1.000** | **0.000** |
| **hyper-boxes** | **HighIC50** | **1.000** | **0.000** | **1.000** | **0.000** | **1.000** | **0.000** |
| Bayes Network | LowIC50 | 1.000 | 1.000 | 1.000 | 1.000 | 1.000 | 1.000 |
|  | HighIC50 | 0.000 | 0.000 | 0.000 | 0.000 | 0.000 | 0.000 |
| Naive Bayes | LowIC50 | 0.769 | 1.000 | 0.885 | 1.000 | 0.577 | 0.667 |
|  | HighIC50 | 0.000 | 0.231 | 0.000 | 0.115 | 0.333 | 0.423 |
| Naive Bayes | LowIC50 | 0.731 | 0.833 | 0.846 | 1.000 | 0.500 | 0.500 |
| Simple | HighIC50 | 0.167 | 0.269 | 0.000 | 0.154 | 0.500 | 0.500 |
| Naive Bayes | LowIC50 | 0.769 | 1.000 | 0.885 | 1.000 | 0.577 | 0.667 |
| Updatable | HighIC50 | 0.000 | 0.231 | 0.000 | 0.115 | 0.333 | 0.423 |
| Lojistic | LowIC50 | 0.846 | 0.833 | 0.692 | 1.000 | 0.731 | 0.833 |
|  | HighIC50 | 0.167 | 0.154 | 0.000 | 0.308 | 0.167 | 0.269 |
| Multilayer | LowIC50 | 0.769 | 1.000 | 0.846 | 0.833 | 0.692 | 0.833 |
| Perceptron | HighIC50 | 0.000 | 0.231 | 0.167 | 0.154 | 0.167 | 0.308 |
| SimpleLogistic | LowIC50 | 0.923 | 1.000 | 0.923 | 1.000 | 1.000 | 1.000 |
|  | HighIC50 | 0.000 | 0.077 | 0.000 | 0.077 | 0.000 | 0.000 |
| SMO | LowIC50 | 1.000 | 1.000 | 1.000 | 1.000 | 1.000 | 1.000 |
| (WEKA SVM) | HighIC50 | 0.000 | 0.000 | 0.000 | 0.000 | 0.000 | 0.000 |
| IB1 | LowIC50 | 0.731 | 1.000 | 0.731 | 1.000 | 0.731 | 0.833 |
|  | HighIC50 | 0.000 | 0.269 | 0.000 | 0.269 | 0.167 | 0.269 |
| IBk | LowIC50 | 0.731 | 1.000 | 0.731 | 1.000 | 0.731 | 0.833 |
|  | HighIC50 | 0.000 | 0.269 | 0.000 | 0.269 | 0.167 | 0.269 |
| Logit Boost | LowIC50 | 0.885 | 1.000 | 0.769 | 1.000 | 0.769 | 1.000 |
|  | HighIC50 | 0.000 | 0.115 | 0.000 | 0.231 | 0.000 | 0.231 |
| Multi Class | LowIC50 | 0.846 | 0.833 | 0.692 | 1.000 | 0.731 | 0.833 |
| Classifier | HighIC50 | 0.167 | 0.154 | 0.000 | 0.308 | 0.167 | 0.269 |
| Threshold | LowIC50 | 0.423 | 0.500 | 0.385 | 0.500 | 0.692 | 0.667 |
| Selector | HighIC50 | 0.500 | 0.577 | 0.500 | 0.615 | 0.333 | 0.308 |
| LMT | LowIC50 | 0.923 | 1.000 | 0.923 | 1.000 | 1.000 | 1.000 |
|  | HighIC50 | 0.000 | 0.077 | 0.000 | 0.077 | 0.000 | 0.000 |
| RandomForest | LowIC50 | 0.885 | 0.833 | 0.846 | 1.000 | 0.808 | 1.000 |
|  | HighIC50 | 0.167 | 0.115 | 0.000 | 0.154 | 0.000 | 0.192 |
| OneR | LowIC50 | 0.923 | 1.000 | 0.885 | 1.000 | 0.923 | 1.000 |
|  | HighIC50 | 0.000 | 0.077 | 0.000 | 0.115 | 0.000 | 0.077 |
